# The psychological mechanisms and behavioral determinants of academic integrity in the age of artificial intelligence

**DOI:** 10.3389/fpsyg.2026.1853790

**Published:** 2026-06-02

**Authors:** Adem Yilmaz

**Affiliations:** Faculty of Education, Department of Mathematics and Science Education, Kastamonu University, Kastamonu, Türkiye

**Keywords:** academic dishonesty, academic procrastination, academic self-efficacy, artificial intelligence use, cheating tendency, learned helplessness

## Abstract

**Objective:**

This study aimed to examine the psychological and behavioral determinants of AI-assisted academic dishonesty among university students through an integrated model. Specifically, the study investigated whether academic procrastination, learned helplessness, and academic self-efficacy predict cheating tendency; whether cheating tendency predicts AI-assisted academic dishonesty; whether AI use moderates the relationship between cheating tendency and academic dishonesty; and whether social and contextual factors significantly predict AI-assisted academic dishonesty.

**Methods:**

The study employed a quantitative, cross-sectional, correlational survey design. A total of 1,045 undergraduate students from different academic disciplines participated voluntarily in the study. Data were collected using seven measurement instruments and a personal information form. Descriptive statistics, Pearson correlation analyses, multiple regression analyses, and Hayes' PROCESS Model 14 were used to test the proposed moderated mediation model. Bootstrap resampling with 5,000 samples was applied to estimate indirect effects and 95% confidence intervals.

**Results:**

The findings showed that academic procrastination and learned helplessness positively predicted cheating tendency, whereas academic self-efficacy negatively predicted it. Cheating tendency significantly predicted AI-assisted academic dishonesty, and the interaction term indicated that the association between cheating tendency and AI-assisted academic dishonesty was stronger at higher levels of AI use. Conditional indirect effect analyses further demonstrated that cheating tendency mediated the effects of academic procrastination, learned helplessness, and academic self-efficacy on AI-assisted academic dishonesty, and these indirect effects became stronger at higher levels of AI use. In addition, social norms, peer behaviors, family attitudes, insufficient sanctions, teacher attitude, high expectations, and adverse conditions significantly predicted AI-assisted academic dishonesty, whereas ethical and moral education emerged as a negative predictor.

**Discussion:**

The findings indicate that AI-assisted academic dishonesty should be understood as a multilevel outcome shaped by the interaction of psychological vulnerabilities, cognitive tendencies, technological affordances, and socio-contextual influences. The study contributes to the academic integrity literature by showing that AI use does not merely accompany dishonest tendencies but amplifies their translation into behavior. These results highlight the need for psychologically informed, ethically grounded, and institutionally supported interventions to reduce academic dishonesty in AI-enhanced higher education environments.

## 1 Introduction

The rapid development of generative artificial intelligence has transformed knowledge production, learning practices, and academic decision-making in higher education. Empirical findings indicate that recent advances in AI technologies have accelerated markedly and have begun to reshape human cognition, learning processes, organizational practices, and behavioral patterns across different domains (Amrullah et al., 2024; Aziz et al., 2025; Coccia, 2025; Ferrarini et al., 2025; Järvelä et al., 2025; Muhammad et al., 2025). Although discussions of AI-driven transformation are sometimes framed through the lens of exponential technological change and critical thresholds, the most immediate relevance of this transformation for higher education lies in the way AI systems increasingly function as cognitive supports in academic work (Femi, 2024; Kwon, 2025). AI tools are no longer used merely for accessing information; they assist students in generating ideas, drafting texts, solving problems, summarizing content, and completing academic tasks (Aguila et al., 2024; Patil and Patil, 2025; Pezzino et al., 2025; Ul Haq, 2025). This transformation has created important opportunities for personalized learning, productivity, and access to academic support. At the same time, it has introduced new ethical challenges by making the boundaries between legitimate assistance, cognitive outsourcing, authorship, and academic misconduct more difficult to distinguish (Bin-Nashwan et al., 2023; Cotton et al., 2024; Gonsalves, 2025; Köbis et al., 2025). One of the most critical consequences of this transformation is the emergence of AI-assisted academic dishonesty. Unlike traditional academic misconduct, AI-assisted dishonesty may occur through practices that are less visible, more difficult to detect, and often perceived by students as ambiguous rather than clearly unethical. For this reason, understanding AI-assisted academic dishonesty requires more than a general discussion of technology use. It requires an explanation of how students' psychological vulnerabilities, cheating-related tendencies, patterns of AI use, and social-contextual conditions are associated with dishonest academic behavior in AI-mediated learning environments.

In this context, AI-assisted academic dishonesty should be understood as a transformed form of academic misconduct shaped by the affordances and ambiguities of generative artificial intelligence. Although academic dishonesty has long been examined in relation to plagiarism, unauthorized collaboration, cheating in examinations, fabrication, and inappropriate citation practices, generative AI has introduced new and more ambiguous forms of misconduct (Bin-Nashwan et al., 2023; Cotton et al., 2024; Gonsalves, 2025). These include submitting AI-generated text as one's own work, using AI systems to paraphrase or conceal copied material, fabricating references or content through generative tools, outsourcing substantial parts of academic assignments to AI, and failing to disclose AI assistance when institutional or course-level rules require transparency (An et al., 2025; Cotton et al., 2024; Gonsalves, 2025). Prior research has shown that generative AI complicates the boundary between legitimate academic support and academic misconduct because students may perceive AI use as assistance, efficiency enhancement, or a morally ambiguous shortcut rather than as a clear violation of academic integrity (Chen et al., 2026; Köbis et al., 2025). However, the existing literature has largely focused on students' general attitudes toward AI, institutional policies, disclosure practices, or ethical concerns (An et al., 2025; Bin-Nashwan et al., 2023; Gonsalves, 2025). Less is known about the psychological and behavioral mechanisms through which students' academic vulnerabilities and cheating-related tendencies are translated into AI-assisted academic dishonesty, particularly under varying levels of AI use and social-contextual influence (Huang et al., 2025; Zanetti and Butera, 2025; Zhao et al., 2025). This gap indicates the need for an integrated explanatory model that examines AI-assisted academic dishonesty not only as a technological issue, but also as a psychologically and socially embedded behavioral outcome.

Conceptually, academic dishonesty refers to intentional or knowingly negligent behaviors that violate the principles of honesty, originality, fairness, transparency, and responsible authorship in academic work. In its conventional forms, academic dishonesty includes plagiarism, cheating during examinations, unauthorized collaboration, fabrication or falsification of data, misrepresentation of sources, and inappropriate citation practices. In AI-mediated academic environments, however, these behaviors are transformed through the affordances of generative technologies. AI-assisted academic dishonesty can therefore be defined as the unauthorized, undisclosed, or misleading use of artificial intelligence tools to produce, modify, conceal, fabricate, or present academic work in ways that violate institutional expectations of academic integrity. This may include submitting AI-generated text as one's own work, using AI tools to paraphrase copied material in order to conceal plagiarism, generating fabricated references or unsupported claims, outsourcing substantial parts of academic assignments to AI systems, using AI during restricted assessments, and failing to disclose AI assistance when transparency is required (Bin-Nashwan et al., 2023; Cotton et al., 2024; Gonsalves, 2025; Köbis et al., 2025). Accordingly, AI-assisted academic dishonesty should be understood not as an entirely new phenomenon detached from earlier forms of misconduct, but as an expanded and technologically mediated form of academic dishonesty in which the boundaries between support, authorship, originality, and misconduct become increasingly difficult to distinguish.

Academic procrastination is defined as the individual's deliberate delay of academic tasks and the continuation of this behavior despite its negative consequences. Kumari (2025) stated that digitalization has changed individuals' task completion habits and that technological tools play an indirect role in this process. However, in the digital age, this behavior has acquired a new dimension. With the widespread use of AI tools, it is observed that individuals postpone their tasks and then turn to technological support at the final stage. This indicates that academic procrastination is no longer merely an individual habit but a dynamic behavior that develops through interaction with technological opportunities (Komajwar, 2025). At this point, the question of the psychological state into which the pressure created by procrastination drives the individual becomes even more critical. The answer to this question lies in the concept of learned helplessness, the second important variable.

Learned helplessness is defined as the condition in which an individual loses the sense of control and ceases to exert effort as a result of repeated experiences of failure. This condition has been shown to be strongly associated, particularly in academic contexts, with loss of motivation, low perceptions of self-efficacy, and declines in performance (Hamal et al., 2025). Indeed, the weakening of an individual's sense of control can directly undermine academic achievement by reducing active participation in learning processes (Kwon, 2025). However, in the age of artificial intelligence, learned helplessness has acquired a different function. The widespread use of digital technologies and artificial intelligence systems leads individuals to turn toward technological tools that can compensate for failure, rather than confronting it directly. In particular, the integration of AI-supported systems into problem-solving and decision-making processes may reduce individuals' cognitive load while simultaneously increasing dependence on external supports (Zhang, 2024). This situation may be associated with a shift in the individual's sense of control from internal processes toward external technological systems. Numerous studies have shown that feelings of academic stress and helplessness direct individuals toward the use of artificial intelligence and that these technologies function as a form of “*psychological compensation mechanism*” (Chen et al., 2025; Liu et al., 2026). The rapid and low-effort solutions offered by artificial intelligence systems may reinforce individuals' avoidance behaviors and, in the long term, deepen the cycle of learned helplessness (Komajwar, 2025). In addition, the excessive use of technology-based solutions may lead to a decline in individuals' problem-solving skills and a weakening of independent thinking processes (Patil and Patil, 2025). This indicates that there is a direct and reciprocal interaction between psychological vulnerability and technological use. At this point, self-efficacy, the third critical variable, comes into play.

Self-efficacy refers to an individual's belief in their ability to successfully accomplish a specific task and is regarded as one of the strongest predictors of academic performance. It is stated that self-efficacy is closely associated with participation in the learning process, academic achievement, and the capacity to carry out tasks independently (Lu et al., 2024). Similarly, it has been reported that as students' AI literacy and their ability to apply these tools increase, their self-efficacy regarding artificial intelligence also rises (Bećirović et al., 2025). The literature shows that individuals with high self-efficacy cope with challenges more effectively and behave in a more controlled manner in their use of technology (Shi et al., 2026; Zhang and Xu, 2025). By contrast, a low level of self-efficacy reduces individuals' confidence in their own effort and facilitates their tendency to turn toward faster, less effortful alternative solutions. In this regard, a study conducted on university students found significant relationships between levels of self-efficacy and self-control and patterns of AI use, and it was stated that more psychologically fragile profiles are particularly more prone to using artificial intelligence in a substitutive manner for academic tasks (Rodríguez-Ruiz et al., 2024). At this point, artificial intelligence comes into play as a tool that reduces the individual's cognitive load while at the same time carrying a risk of dependency. In particular, studies examining the relationships among academic self-efficacy, academic stress, performance expectations, and AI dependency (Acosta-Enriquez et al., 2025; Liu et al., 2026) have indicated that low academic self-efficacy may indirectly foster AI dependency, which in turn may produce negative consequences for creativity, critical thinking, and independent thinking (Zhang et al., 2024). Therefore, artificial intelligence may be regarded not only as a supportive learning tool but also as a mechanism that may become susceptible to excessive use when an individual's psychological resources are weak. Accordingly, self-efficacy may be considered a critical psychological filter that determines how the individual uses technology. While individuals with high self-efficacy tend to use artificial intelligence as a complementary support, individuals with low self-efficacy appear more likely to turn to these tools in a more compensatory and dependency-producing manner. A similar pattern also emerges in the ethical dimension.

In the context of AI use, strengthening self-efficacy regarding academic integrity and appropriate use is considered a protective factor that may reduce unethical tendencies (Creighton et al., 2025). The interaction of these three fundamental variables does not necessarily correspond directly to observable behavior. Rather, it may first be reflected in a cheating-related tendency. The behavioral sciences literature demonstrates that individuals' behaviors are shaped through cognitive and emotional processes and that these processes generate pre-behavioral intentions and tendencies (Huang et al., 2025; Izquierdo-Condoy et al., 2025). At this point, the concept of cheating tendency emerges. In the academic context, this tendency manifests itself as an internal evaluative process regarding the extent to which an individual may stretch ethical boundaries. In particular, the widespread use of artificial intelligence has created a new context in which this tendency may become more salient. Studies on AI-supported learning environments (O'Dea et al., 2025; Rizun et al., 2026) show that students perceive these technologies as both supportive tools and tools that potentially carry ethical risks (Fawaz et al., 2025). At the same time, it is stated that the conveniences provided by AI use in academic writing and production processes may indirectly influence individuals' ethical decision-making processes and may create a kind of cognitive legitimization process prior to behavior (Jojua, 2025). However, the tendency does not always turn into behavior. This transformation emerges under certain conditions, and the technological context in which the individual is situated plays a determining role in this process. Indeed, there are findings indicating that in situations where AI use intensifies, individuals' perceptions of academic integrity may weaken and their tendency toward unethical behavior may increase (Lam et al., 2025). At this point, AI use assumes a critical role in the process as a moderating variable. Artificial intelligence may function as a tool that is associated with a stronger link between an individual's cheating tendency and dishonest behavior. In particular, it is emphasized that excessive dependence on artificial intelligence may weaken critical thinking and independent production processes, thereby increasing violations of academic integrity (Arhin, 2025). The fundamental reason for this is that artificial intelligence provides the individual with rapid, low-cost, and high-quality outputs. This situation facilitates the stretching of ethical boundaries and lowers the individual's threshold for acting on such behavior. Therefore, artificial intelligence may be regarded not merely as a tool, but also as a contextual condition associated with students' cognitive evaluation processes and the enactment of dishonest academic behavior.

Finally, academic dishonesty behavior, which constitutes the outcome of this multilayered process, is not merely the result of individual psychological factors. It is shaped under the influence of social and environmental factors. It is stated that individuals' ethical decisions are strongly influenced by the social context and that peer influence, institutional norms, and learning environments are particularly determinant in this process (Lei et al., 2025). Similarly, it is emphasized that the social norms perceived in academic environments constitute one of the fundamental reference points guiding an individual's ethical behavior (Bozkurt et al., 2025). Social norms, peer behaviors, family attitudes, teacher approaches, and sanction mechanisms are contextual factors that directly affect the individual's ethical decisions. Studies conducted particularly in AI-supported learning environments show that students develop attitudes toward these technologies by being influenced by the ways in which they are used in their surroundings, and that this is reflected in ethical decision-making processes (Abdelwahab et al., 2025). In this context, environments in which peers normalize academic integrity violations may be associated with a higher likelihood of dishonest behavior, whereas environments characterized by strong ethical norms and monitoring mechanisms may be associated with a lower likelihood of such behavior (Erliani et al., 2025). Within this framework, academic dishonesty should be regarded as a multilayered behavior emerging at the intersection of individual psychological variables, cognitive tendencies, technological tools, and social environmental factors. Indeed, systematic reviews of AI-supported learning environments show that these technologies transform not only cognitive processes but also attitudes, ethical perceptions, and behavioral patterns (Bozkurt et al., 2025; Park, 2025; Wu et al., 2025). Accordingly, psychological variables such as academic procrastination, learned helplessness, and self-efficacy may be associated with cheating tendency, and this tendency may be more strongly linked to AI-assisted academic dishonesty under higher levels of AI use, while social norms and environmental factors may also be related to this behavior. This holistic structure suggests that academic integrity violations should be addressed not merely as an individual problem but as a socio-technological phenomenon. In this regard, the present study aims to examine the psychological, behavioral, technological, and socio-contextual correlates of AI-assisted academic dishonesty within the framework of an integrated model.

### 1.1 Theoretical framework

The present study is theoretically informed by the Stimulus–Organism–Response (SOR) framework, which provides a useful explanatory structure for understanding how external and internal conditions are translated into behavioral outcomes through intervening cognitive and affective mechanisms (Mehrabian and Russell, 1974). In the context of AI-assisted academic dishonesty, psychological vulnerabilities and socio-contextual conditions can be conceptualized as stimuli that shape students' internal evaluations of academic tasks, ethical boundaries, and available behavioral options. Academic procrastination, learned helplessness, and low academic self-efficacy represent internal psychological stimuli that may increase students' cognitive openness to dishonest alternatives, whereas peer behaviors, social norms, family attitudes, institutional sanctions, teacher attitudes, and performance expectations represent contextual stimuli that define the perceived acceptability or risk of such behaviors (Huang et al., 2025; Zanetti and Butera, 2025; Zhao et al., 2025). Within this framework, cheating tendency functions as the organismic mechanism through which these stimuli are cognitively and motivationally processed before being translated into behavior. AI-assisted academic dishonesty, in turn, represents the behavioral response that may emerge when cheating tendency is activated under conditions of technological accessibility and ethical ambiguity. AI use is positioned as a technological condition that may intensify the association between cheating tendency and dishonest behavior by reducing effort costs, increasing task automation, and blurring the boundary between legitimate support and misconduct (Cotton et al., 2024; Gonsalves, 2025; Köbis et al., 2025). To reduce overlap with the empirical literature review, this section focuses on the theoretical positioning of the study variables within the proposed model. The empirical evidence supporting each construct is discussed separately in the Literature Review section. Therefore, the SOR framework is well aligned with the proposed moderated mediation model, as it explains how psychological and contextual stimuli may be linked to AI-assisted academic dishonesty through cheating tendency, and why this association may become stronger at higher levels of AI use.

#### 1.1.1 Academic procrastination as a self-regulatory breakdown

Academic procrastination is conceptualized in the present model as a self-regulatory vulnerability rather than merely a deficiency in time management. It reflects cognitive avoidance, emotional dysregulation, motivational fluctuation, and a tendency to prioritize short-term emotional relief over long-term academic goals (Zhang et al., 2024). In higher education, this vulnerability becomes more salient under academic stress and cognitive load, where students' self-regulatory capacity may weaken and avoidance-based coping strategies may become more likely (Bećirović et al., 2025). In digitally enriched and AI-supported learning environments, procrastination also acquires a techno-cognitive dimension: delayed academic tasks may be completed through rapid technological assistance, making AI tools attractive as low-effort compensatory resources (Rodríguez-Ruiz et al., 2024). Findings on AI-supported learning environments further suggest that excessive reliance on automated systems may be associated with avoidance of cognitive effort and weaker self-regulatory engagement (Lei et al., 2025). Within the proposed model, academic procrastination is therefore positioned as an initial psychological antecedent that may be positively associated with cheating tendency by increasing students' openness to easier, faster, or ethically ambiguous academic alternatives.

#### 1.1.2 Learned helplessness and adaptive disengagement in AI contexts

Learned helplessness is positioned in the present model as a psychological condition associated with reduced perceived control, lower persistence, avoidance tendencies, and greater reliance on external support systems in academic task completion (Zhang et al., 2024). In AI-integrated learning environments, this condition may acquire a technological dimension because students experiencing helplessness may perceive AI tools as substitutes for their own academic competencies rather than as complementary learning supports. In this sense, learned helplessness can be interpreted as a form of adaptive disengagement in which students withdraw from effortful learning processes and become more likely to delegate cognitive tasks to intelligent systems (Dwivedi et al., 2023). Research on AI-supported learning further suggests that low self-regulation and psychological vulnerability may be associated with the easier legitimization of AI use in ethically ambiguous situations, potentially reducing cognitive dissonance around problematic academic behaviors (Dwivedi et al., 2023; Kasneci et al., 2023). Within the proposed model, learned helplessness is therefore not treated as a direct cause of academic dishonesty, but as a psychological antecedent that may be positively associated with cheating tendency by reshaping students' perceived control, effort expectations, and internal thresholds of acceptability.

#### 1.1.3 Self-efficacy as a regulatory filter in AI-enhanced environments

Academic self-efficacy refers to students' beliefs in their capacity to successfully perform academic tasks and is positioned in the present model as a regulatory filter shaping effort, coping strategies, and ethically relevant academic decisions (Chun et al., 2025; Schunk and DiBenedetto, 2020; Wang, 2026). In AI-enhanced learning environments, this regulatory function becomes more complex because AI tools may simultaneously support academic task completion and increase reliance on external cognitive assistance (Zhang and Xu, 2025). From this perspective, self-efficacy may shape whether students use AI as a complementary learning support or as a substitute for their own academic effort (Nguyen et al., 2020; Zhang et al., 2025). On the one hand, AI-supported task completion may be associated with stronger perceived competence; on the other hand, intensive reliance on external support may be linked to cognitive externalization and weaker confidence in one's own academic capacity (Shi et al., 2026). Within the proposed model, academic self-efficacy is therefore conceptualized as a protective psychological factor that may be negatively associated with cheating tendency, particularly because students with stronger self-efficacy are expected to rely more on their own effort and to interpret ethical boundaries more cautiously in AI-mediated academic contexts.

#### 1.1.4 Cheating tendency as a cognitive-behavioral mediator

The movement from psychological vulnerability to observable academic behavior does not necessarily occur directly; rather, it may be shaped by cognitive and motivational processes that reflect students' readiness to engage in ethically problematic conduct. In the present model, this intermediary construct is conceptualized as cheating tendency, referring to students' implicit predisposition toward stretching academic integrity boundaries. Current studies suggest that cheating tendency may be associated with perceived task difficulty, emotional stress, and the availability of alternative solution pathways (Fawaz et al., 2025). In AI-mediated learning environments, this tendency becomes theoretically important because generative AI tools provide rapid, low-cost, and high-quality outputs that may reduce the perceived effort and psychological barriers associated with unethical academic behavior (Dogaru et al., 2025; Seran et al., 2025). At the same time, cheating tendency should not be treated solely as an individual trait, because it may also be shaped by technological norms and institutional practices (Chavez et al., 2024). Within the proposed model, cheating tendency is therefore positioned as a cognitive-behavioral mediator that links psychological antecedents to AI-assisted academic dishonesty by representing the evaluative readiness through which students interpret ethically ambiguous academic options.

#### 1.1.5 Artificial intelligence use as a behavioral amplifier (moderator)

Artificial intelligence use is positioned in the present model as a contextual moderator that may alter the strength of the association between cheating tendency and AI-assisted academic dishonesty. Unlike traditional academic tools, generative AI systems can reshape the time, effort, and task-execution conditions involved in academic production by supporting learning in some contexts while also enabling cognitive outsourcing and more instrumental or surface-level engagement in others (Bin-Nashwan et al., 2023; Liu et al., 2024; Montag and Elhai, 2025). Importantly, AI use is not treated here as a direct cause of unethical behavior; rather, it is conceptualized as a technological condition that may make the enactment of pre-existing cheating tendencies easier when effort costs are low, task delegation is possible, and institutional boundaries are ambiguous. Studies suggest that generative AI can make cheating more accessible and that students may evaluate explicit violations and “gray-area” practices differently in AI-mediated academic contexts (Köbis et al., 2025). Moreover, nondisclosure of AI use, blurred boundaries between assistance and misconduct, and weakened assumptions of originality in assessment practices further support the moderating role of AI use in this model (Cotton et al., 2024; Gonsalves, 2025). Therefore, AI use is conceptualized as a behavioral amplifier in a non-causal statistical sense: it may be associated with a stronger link between cheating tendency and AI-assisted academic dishonesty, particularly when ethical boundaries are perceived as uncertain (Huang et al., 2025; Köbis et al., 2025).

#### 1.1.6 Academic dishonesty as a multilevel behavioral outcome

Academic dishonesty is conceptualized in the present model as a multilevel behavioral outcome associated with the interaction of psychological vulnerabilities, technological affordances, social norms, and institutional conditions. The collective cheating culture approach suggests that dishonest behavior is not only related to individual characteristics but also to descriptive norms embedded in academic environments (Zanetti and Butera, 2025). In the context of GenAI, students' cheating-related behaviors may also be associated with peer influence, classroom-level practices, ambiguous guidelines, inconsistent perceptions of sanctions, and social pressure (Gonsalves, 2025; Huang et al., 2025). These contextual dynamics become particularly salient in AI-supported environments, where institutional policies toward generative AI remain inconsistent and where the boundary between legitimate assistance and academic misconduct is increasingly blurred (An et al., 2025; Cotton et al., 2024). Moreover, assessment practices based solely on assumptions of originality and authenticity may be insufficient as protective mechanisms in the face of generative AI (Kofinas et al., 2025). Within this multilevel perspective, AI-assisted academic dishonesty is positioned as the outcome of a broader socio-technological process in which psychological antecedents, cheating tendency, AI use, social norms, and institutional expectations are jointly considered. Accordingly, the present study proposes an integrated model in which academic procrastination, learned helplessness, and academic self-efficacy are treated as psychological antecedents; cheating tendency is treated as the proximal cognitive-behavioral mediator; AI use is treated as a moderator; and social-contextual factors are examined as additional correlates of AI-assisted academic dishonesty.

Within this framework, cheating tendency is conceptualized, in a manner consistent with contemporary behavioral intention models adapted to digital learning environments, as a mediating construct that transforms internal psychological states into behavioral outcomes. Indeed, the model proposed by Huang et al. (2025) in the context of generative artificial intelligence demonstrates that students' ethical evaluations and cheating tendencies operate as proximal cognitive mechanisms directly leading to academic dishonesty. However, the present model advances this perspective by incorporating AI use as a moderating variable, particularly one that affects the strength of the relationship between cheating tendency and academic dishonesty. This assumption is consistent with the findings of Kofinas et al. (2025), which show that generative artificial intelligence weakens integrity thresholds in assessment processes, as well as with the experimental results of Köbis et al. (2025), which reveal that delegating tasks to artificial intelligence can reduce the perceived moral cost of unethical behavior.

**Figure 1:**
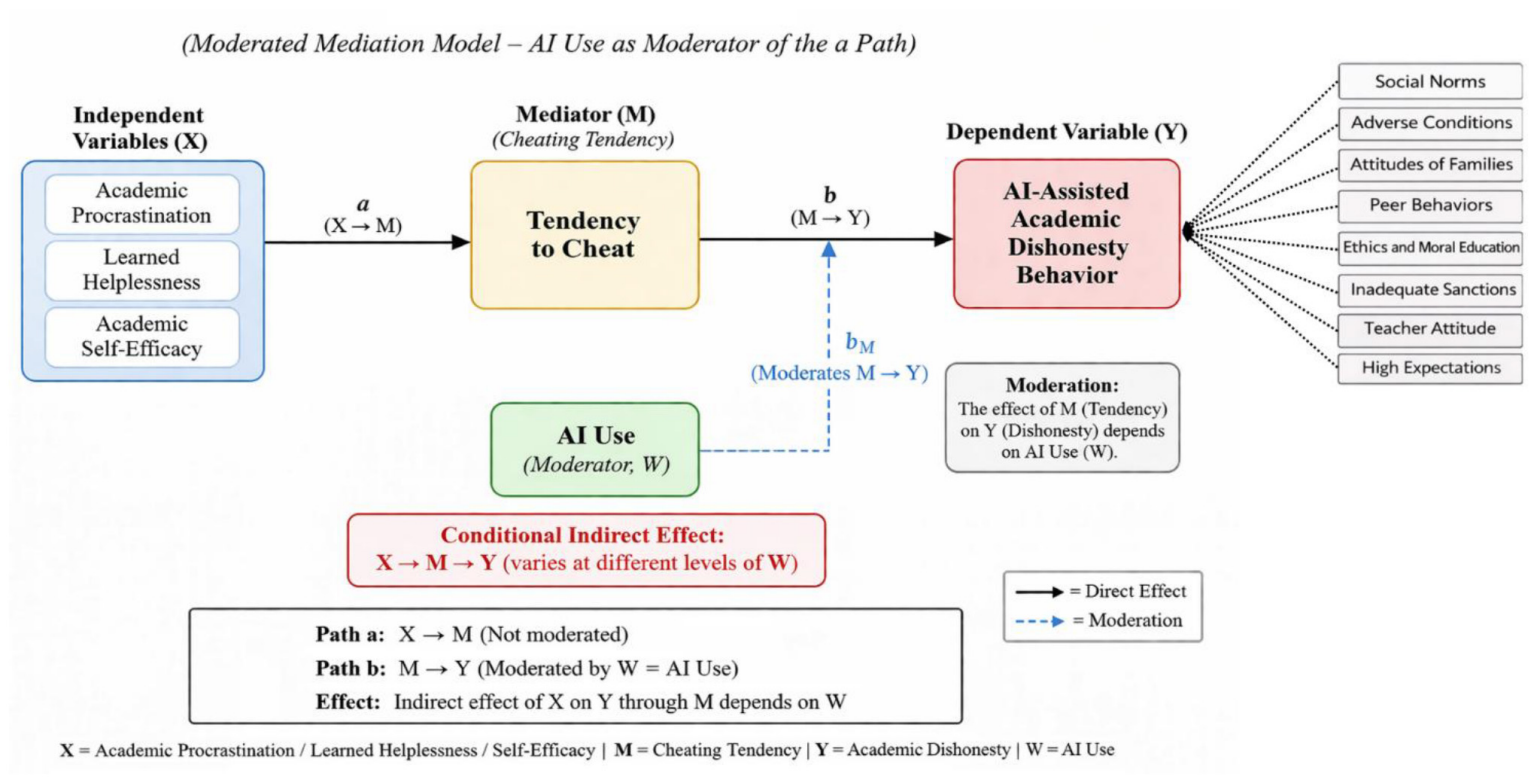
Research model.

In other words, as the accessibility and functional capacity of AI tools increase, the likelihood that intentions to violate academic integrity—previously remaining only at the level of tendency—will be transformed into behavior also increases. On the other hand, the model also acknowledges that academic dishonesty does not emerge in a vacuum; rather, it is embedded within a broader socio-educational context. For this reason, social norms, peer behaviors, family attitudes, institutional sanctions, teacher approaches, and performance expectations are treated as external determinants of the framework. This approach is supported by Zanetti and Butera's (2025) study, which conceptualizes collective cheating culture in academic settings as a descriptive norm, as well as by the meta-analysis of Zhao et al. (2025), covering 80 studies from 27 countries and 40,867 participants, which demonstrates that cultural values significantly shape the patterns of academic cheating behavior. Similarly, while Gonsalves (2025) reveals that ambiguous guidelines and inconsistent perceptions of sanctions weaken the disclosure of AI use, An et al. (2025) show that the institutional climate and explicit course-level policies are determinant in how students interpret the use of artificial intelligence. Therefore, academic dishonesty should be explained not only through the individual's internal characteristics, but also together with the normative and institutional environment within which the individual operates. Considered holistically, the proposed model offers a comprehensive and theoretically grounded framework that integrates individual psychological processes, cognitive mediation mechanisms, technological affordances, and contextual influences within a single analytical structure. In this respect, the model goes beyond fragmented explanations and makes it possible to understand academic dishonesty more holistically in the age of artificial intelligence. In the following section, built upon this conceptual foundation, the empirical and theoretical literature concerning each component of the model is examined in detail, and psychological antecedents, mediating mechanisms, moderating effects, and contextual determinants are discussed systematically. The research model is presented in Figure 1.

### 1.2 Literature review

#### 1.2.1 Academic procrastination

Academic procrastination is a common problem defined as the student's delay of academic tasks despite knowing their negative consequences, and it is closely associated with disruptions in self-regulation processes. The systematic review by Salguero-Pazos and Reyes-de-Cózar (2023) demonstrates that academic procrastination cannot be explained solely by a lack of time management; rather, it must be addressed together with self-regulation deficiencies, anxiety, personality traits, and motivational vulnerabilities. Similarly, in their review synthesizing 27 empirical studies, Ramadhani et al. (2026) show that fear, perfectionism, low motivation, and difficulties in emotional regulation are among the main determinants of academic procrastination. On a more contextual level, Torrens et al. (2026) have shown that task complexity, simultaneous workload, and a low sense of confidence drive students toward chronic procrastination behavior. Therefore, academic procrastination should be regarded not as a simple habit of delay, but as a multidimensional process in which cognitive load, emotional strain, and weaknesses in self-regulation converge.

With the widespread use of generative artificial intelligence tools, this process has acquired a new dimension. Although high-quality studies directly examining the relationship between AI and academic procrastination are still limited, existing findings suggest that these tools may serve a “*compensatory”* function, particularly for students under pressure. While Zhang et al. (2024) show that low academic self-efficacy and academic stress indirectly pave the way for problematic AI use, Zhang and Xu (2025) demonstrate that the frequency of generative AI use can simultaneously increase both the sense of self-efficacy and technological dependence in the task completion process. These findings suggest that, because procrastination directs individuals under time pressure toward faster and lower-effort solutions, artificial intelligence may become an enabler that indirectly reinforces procrastination behavior. Therefore, academic procrastination should be addressed not so much as a direct producer of unethical behavior, but rather as an initial condition that is associated with higher levels of cognitive openness toward such behaviors.

#### 1.2.2 Learned helplessness

Learned helplessness is conceptualized as the condition in which an individual, following repeated experiences of failure or loss of control, comes to believe that their effort will not produce results and therefore withdraws from effective coping behaviors. Recent studies clearly demonstrate that this construct weakens academic engagement and learning performance. For example, Azad and Yavuz (2025), in their study conducted with university students, showed that learned helplessness is negatively associated with learning outcomes. Similarly, Liu et al. (2024) found that when students experience effort–reward imbalance, their levels of learned helplessness increase, which in turn significantly reduces learning engagement. The same study also showed that social support partially buffers this negative effect. These findings indicate that learned helplessness not only creates a loss of motivation, but also weakens the cognitive and emotional relationship the individual establishes with the task.

In the context of artificial intelligence, direct evidence testing the “*learned helplessness–AI use*” connection remains limited; however, the adjacent literature offers important insights. Zhang et al. (2024) showed that low academic self-efficacy and high academic stress strengthen the pathway toward AI dependency, while Acosta-Enriquez et al. (2025) demonstrated that academic self-efficacy, stress, and performance expectations together explain students' AI dependency. These findings suggest that students whose sense of control is weakened and who are unable to manage academic difficulties through their internal resources may externalize problem-solving and use artificial intelligence less as a support tool and more as a substitutive mechanism. Therefore, in the age of artificial intelligence, learned helplessness may be regarded not merely as a passive emotional state, but as a risk structure that can direct students toward external, rapid, and cognitively load-reducing solutions.

#### 1.2.3 Academic self-efficacy

Academic self-efficacy refers to the student's belief in their ability to successfully carry out academic tasks and is regarded as one of the strongest psychological determinants of the learning process. The current literature shows that high self-efficacy is associated not only with better performance, but also with more stable self-regulation and more resilient coping strategies. In the age of artificial intelligence, however, the relationship between self-efficacy and technology use is no longer linear. Zhang and Xu (2025) show that the use of generative artificial intelligence can, on the one hand, increase students' confidence in the task completion process, while, on the other hand, also fostering technological dependence. In parallel, Zhang et al. (2024) demonstrated that low self-efficacy does not by itself explain AI dependency; however, it creates an indirect risk pathway through academic stress and performance expectations.

This dual structure indicates that self-efficacy should now be regarded not merely as an individual belief system, but as a dynamic construct shaped through interaction with technology. Indeed, the findings of Acosta-Enriquez et al. (2025) show that the effect of academic self-efficacy on AI dependency is significantly mediated by academic stress and performance expectations. In other words, even if students with low self-efficacy do not turn directly to artificial intelligence, they may rely on it more heavily when they feel under pressure. For this reason, low academic self-efficacy may predispose students not only to avoid academic tasks, but also to transform artificial intelligence from a supportive tool into a substitutive solution mechanism. Within this framework, when academic procrastination, learned helplessness, and self-efficacy are considered together, a powerful psychological foundation emerges that determines how students respond to academic processes; however, this foundation produces not behavior directly, but rather a tendency in the first instance.

#### 1.2.4 Cheating tendency

Cheating tendency may be regarded as the proximal mechanism of cognitive and emotional preparation that directly precedes academic dishonesty itself. For this reason, in order to understand the final form of the behavior, it is necessary to focus not only on the question of “*who cheated?”* but also on the conditions under which the student begins to perceive this behavior as legitimate or feasible. In the extended Theory of Planned Behavior model they developed in the context of generative artificial intelligence, Huang et al. (2025) demonstrate that students' moral evaluations, norm perceptions, and behavioral intentions constitute proximal cognitive pathways leading to GenAI-assisted cheating behavior. Similarly, the study by Reiter et al. (2025), by jointly examining the willingness to use AI for university-related tasks and the prevalence of unauthorized AI use in examinations, reveals that the relationship between intention to use and the stretching of ethical boundaries is shaped not only by technical acceptance but also by perceptions of opportunity and trust.

In AI-supported environments, this tendency may become even stronger because of the speed, accessibility, and low effort cost provided by the tool. Köbis et al. (2025) have shown that delegating tasks to artificial intelligence can reduce the perceived moral cost of dishonest behaviors and that this can increase unethical behaviors. This finding is important because it shows that cheating tendency is shaped not only by the individual's personal moral weakness, but also by the technological setting in which the individual is situated. Therefore, cheating tendency should be regarded not as a fixed trait nourished by individual variables, but as a dynamic intermediary mechanism that is strengthened by task difficulty, time pressure, normative ambiguity, and technological convenience. The transformation of this tendency into actual behavior generally occurs through facilitators such as artificial intelligence, which make the behavior less costly.

#### 1.2.5 AI use

In this model, AI use is treated as a critical moderating variable that influences the process through which an individual's existing tendency is transformed into behavior. Generative artificial intelligence systems not only make it possible to complete tasks more quickly, but also transform the very nature of academic labor. Bin-Nashwan et al. (2023) emphasize that the opportunities created by artificial intelligence in the academic context give rise to serious tensions in terms of academic integrity. In parallel, Kim et al. (2025) showed that students' and faculty members' attitudes toward generative artificial intelligence converge across many dimensions; however, students find artificial intelligence easier to use and more appealing. This finding indicates that AI use is shaped not only by technical access, but also by perceived ease and usage comfort.

In addition, evidence is increasing that AI use reshapes students' regulation of cognitive effort. Zhang and Xu (2025) showed that more frequent use of generative artificial intelligence, while capable of supporting self-efficacy, can also increase technological dependence. Chen et al. (2026), in turn, report that students use artificial intelligence intensively particularly for supportive tasks such as explaining concepts, generating ideas, and summarizing, whereas they draw more cautious boundaries in areas of clear violation, such as completing an entire assignment. This suggests that AI use transforms students' perceptions of what constitutes “*assistance*” and what constitutes “*misconduct*”. Therefore, artificial intelligence is not merely a tool; it is a force that restructures academic decision-making, cognitive labor, and the interpretation of ethical boundaries. For this reason, it assumes a decisive moderating role in the transformation of cheating tendency into behavior.

#### 1.2.6 Academic dishonesty

Academic dishonesty is a multilayered behavior that emerges from the interaction of individual psychological processes, technological affordances, and the social context. For this reason, explaining academic misconduct solely in terms of individual moral weakness is insufficient. The “*collective cheating culture approach*” developed by Zanetti and Butera (2025) shows that students may experience integrity violations not only as a personal decision but also as a collective practice that can become normalized within the peer group. On a broader level, the meta-analysis by Zhao et al. (2025), which includes 80 studies from 27 countries, demonstrated that cultural values and normative patterns significantly shape academic cheating behavior. These findings confirm that external factors such as peer behavior, family attitudes, and institutional climate strongly influence ethical decisions.

In AI-supported environments, this multilevel structure becomes even more pronounced. An et al. (2025) showed that there are significant differences in the generative artificial intelligence policies of higher education institutions, and that these differences affect how students interpret the boundaries of acceptable use. Gonsalves (2025), on the other hand, revealed that students' low compliance with AI-use disclosure is closely associated with ambiguous guidelines and inconsistent perceptions of sanctions. Chen et al. (2026), meanwhile, showed that overall cheating rates may remain stable; however, students are reframing which tasks are considered “*appropriate*” when using artificial intelligence. Therefore, academic dishonesty should be conceptualized as a complex behavioral outcome that emerges at the intersection of individual tendencies, social norms, and technological affordances.

### 1.3 Purpose of the study and hypotheses

The main purpose of this study is to examine the psychological and behavioral mechanisms underlying academic integrity violations in the age of artificial intelligence within the framework of a holistic model. In this regard, the effects of individual psychological variables such as academic procrastination, learned helplessness, and academic self-efficacy on individuals' cheating tendency are analyzed, and the manner in which this tendency is transformed into academic dishonesty behavior is investigated. In addition, the study examines how AI use, by playing a moderating role in this process, affects the likelihood that individuals' existing tendencies will be transformed into behavior. At the same time, social norms and contextual factors are incorporated into the model in order to provide a multilayered explanation of academic integrity. In line with this purpose, the following hypotheses have been developed:

***H1:*** *Academic procrastination is significantly and positively associated with cheating tendency*.
***H2:*** *Learned helplessness is significantly and positively associated with cheating tendency*.
***H3:*** *Academic self-efficacy is significantly and negatively associated with cheating tendency*.
***H4:*** *Cheating tendency is significantly and positively associated with AI-assisted academic dishonesty behavior*.
***H5:*** *Academic procrastination is significantly and positively associated with AI-assisted academic dishonesty behavior*.
***H6:*** *Learned helplessness is significantly and positively associated with AI-assisted academic dishonesty behavior*.
***H7:*** *Academic self-efficacy is significantly and negatively associated with AI-assisted academic dishonesty behavior*.
***H8:*** *Cheating tendency mediates the association between academic procrastination and AI-assisted academic dishonesty behavior*.
***H9:*** *Cheating tendency mediates the association between learned helplessness and AI-assisted academic dishonesty behavior*.
***H10:*** *Cheating tendency mediates the association between academic self-efficacy and AI-assisted academic dishonesty behavior*.
***H11:*** *AI use moderates the association between cheating tendency and academic dishonesty behavior; this association is expected to be stronger at higher levels of AI use*.
***H12:*** *Social norms, peer behaviors, family attitudes, ethical and moral education, insufficient sanctions, teacher attitude, and high expectations are significantly associated with AI-assisted academic dishonesty behavior*.

**Figure 2:**
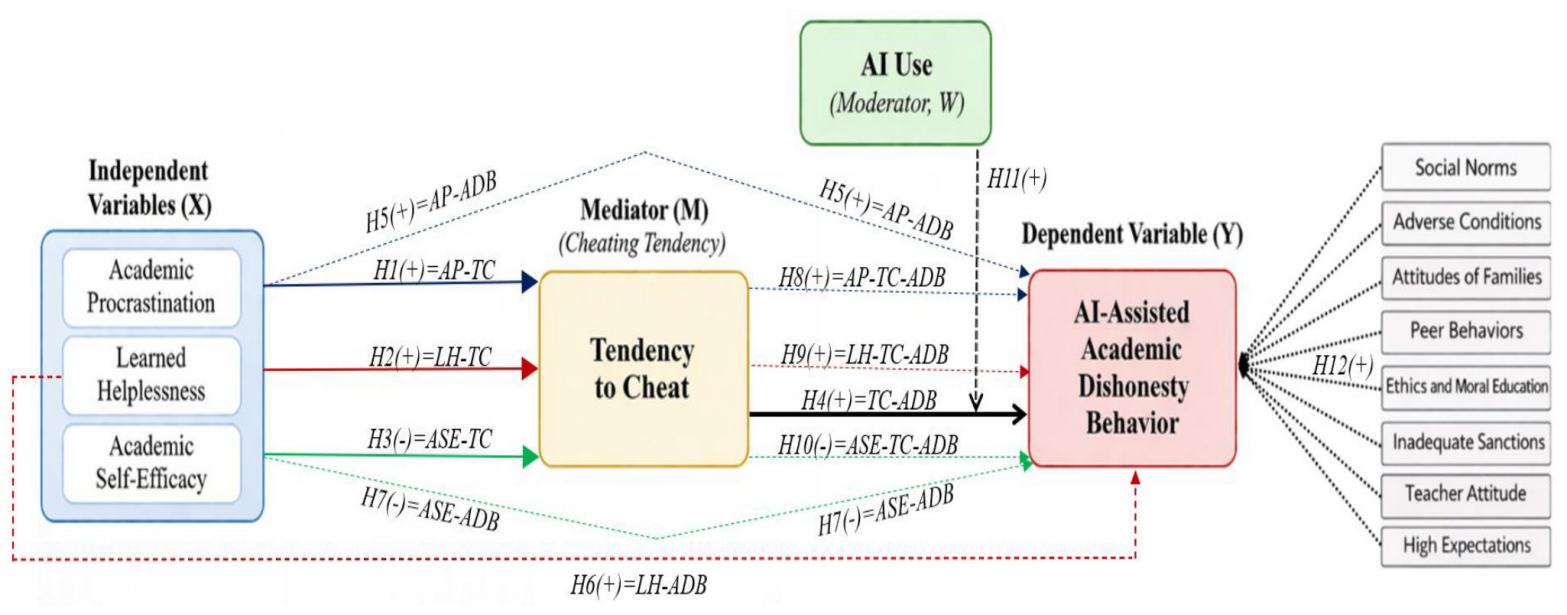
Hypotheses.

The hypotheses presented in Figure 2 illustrate how academic procrastination, learned helplessness, and academic self-efficacy influence academic dishonesty behavior through cheating tendency, while also highlighting the moderating role of AI use in this process. In addition, the model incorporates the direct effects of social and contextual factors on academic dishonesty, offering a more comprehensive framework for understanding this behavior.

## 2 Materials and methods

### 2.1 Research design

The present study employed a quantitative, cross-sectional, correlational survey design to examine the psychological and behavioral mechanisms underlying AI-assisted academic dishonesty among university students (Creswell and Creswell, 2018; Setia, 2016). This design was selected because it allows the investigation of relationships among the main research variables—namely academic procrastination, learned helplessness, academic self-efficacy, cheating tendency, AI use, and academic dishonesty—within a natural higher education context (Fraenkel et al., 2019; Setia, 2016). Data were collected during the regular academic semester through structured self-report questionnaires administered to undergraduate students from different academic disciplines. Since the study aimed to analyze naturally occurring differences in students' psychological characteristics, technology use, and integrity-related behaviors rather than to manipulate these variables experimentally, a cross-sectional survey approach was considered methodologically appropriate (Creswell and Creswell, 2018; Fraenkel et al., 2019). The study context was shaped by routine academic practices, formal assessment processes, and increasing exposure to AI-supported learning tools; this provided an appropriate setting for examining the interaction of psychological predispositions, cognitive tendencies, technological affordances, and contextual influences in the emergence of academic dishonesty.

### 2.2 Participants

A total of 1,045 undergraduate students voluntarily participated in the study. The sample consisted of full-time university students enrolled in different academic programs, including engineering, education, health sciences, economics and administrative sciences, fine arts, and vocational school programs. Participants ranged in age from 17 to 24 years, with a mean age of 20.63 years (SD = 1.84). The sample included both female and male students and represented different year levels of undergraduate education. In order to increase the diversity of academic backgrounds, students from multiple disciplinary fields were included in the study. This heterogeneous composition was considered important for obtaining a broader picture of AI-assisted academic dishonesty within the higher education context. Participants were recruited using a convenience sampling strategy through classroom announcements and institutional communication channels. Participation was entirely voluntary, and no financial or academic incentives were provided. All respondents were informed about the purpose of the study and participated on the basis of informed consent. The relatively balanced distribution across gender, department, and grade level enhanced the representativeness of the sample within the study context.

### 2.3 Measures

A total of seven measurement instruments and an additional personal information form were used to assess the main variables of the study. Specifically, academic procrastination, learned helplessness, academic self-efficacy, cheating tendency, AI use, social and contextual factors, and academic dishonesty were measured through the instruments included in the study. Among these, the scales measuring academic procrastination, learned helplessness, academic self-efficacy, and academic dishonesty were based on previously developed instruments, whereas the measures of cheating tendency, AI use, and social and contextual factors were developed by the researchers in line with the theoretical framework of the study. In addition, a brief form was used to collect participants' demographic information. Previously established scales were administered in their Turkish forms, and the researcher-developed instruments were prepared in Turkish for the purposes of the present study. Prior to the main analyses, the internal consistency and construct validity of each instrument were re-examined within the present sample.

#### 2.3.1 Academic procrastination (AP)

Academic procrastination was assessed using the Procrastination Assessment Scale–Students (AP) developed by Solomon and Rothblum (1984). The AP is a 44-item self-report instrument rated on a 5-point scale and organized into two sections. The first section assesses students' procrastination tendencies across six academic areas, namely writing a term paper, studying for an exam, keeping up with weekly reading assignments, performing administrative tasks, attending meetings, and carrying out academic tasks in general. The second section evaluates the possible reasons underlying procrastination behavior. In the present study, the scale was used to assess participants' level of academic procrastination within the context of higher education.

#### 2.3.2 Learned helplessness (LH)

Learned helplessness was assessed using the Learned Helplessness Scale adapted by Lan et al. (2024) for Chinese law school students. In the present study, the 14-item version of the scale was adopted and translated into Turkish for research purposes. The scale is a self-report instrument designed to assess individuals' levels of learned helplessness, and participants respond to the items on a 4-point Likert-type scale. Response options range from 1 = strongly agree to 4 = strongly disagree. In the process of constructing the Turkish form of the scale, careful attention was given to translation and adaptation procedures, and its validity and reliability were re-examined within the present sample. In the adaptation study conducted with the Chinese sample, the four-factor, 14-item structure of the scale was reported to demonstrate acceptable psychometric properties.

#### 2.3.3 Academic self-efficacy (ASE)

Academic self-efficacy was assessed using the Academic Self-Efficacy Scale (ASE), the validity and reliability of which were examined by Hemade et al. (2025) among Arabic-speaking university students. In the present study, the 9-item version of the scale was adopted and translated into Turkish for research purposes. The scale is a self-report instrument designed to assess students' perceptions of their competence in performing academic tasks. The items cover self-evaluations related to academic skills such as time management, note-taking, studying for examinations, conducting research, writing academic papers, and succeeding at university. Participants respond to the items on a 7-point Likert-type scale, with response options ranging from 1 = strongly disagree to 7 = strongly agree. Higher scores on the scale indicate higher levels of academic self-efficacy. In the process of constructing the Turkish form of the scale, careful attention was given to translation and adaptation procedures, and its validity and reliability were re-examined within the present sample. In the Arabic adaptation study, the one-factor, 9-item structure of the scale was confirmed and reported to demonstrate high internal consistency.

#### 2.3.4 Cheating tendency (TC)

Cheating tendency was assessed using the cheating tendency (TC) developed for the purposes of the present study. The item pool was generated on the basis of the relevant literature and the theoretical framework of the study, which conceptualized cheating tendency as a proximal cognitive predisposition toward academic dishonesty. The preliminary form of the scale consisted of 20 items covering four dimensions: tendency to stretch ethical boundaries, opportunity- and convenience-oriented tendency, justification under pressure, and AI-assisted cheating tendency. Participants responded to the items on a 5-point Likert-type scale, ranging from 1 = strongly disagree to 5 = strongly agree. Higher scores on the scale indicated higher levels of cheating tendency. Prior to the main analyses, the wording and content relevance of the items were reviewed, and the psychometric properties of the scale were examined within the present sample.

#### 2.3.5 AI use (AIUS)

AI use was assessed using the AI Use Scale (AIUS) developed by the researcher for the purposes of the present study. The scale was designed to measure the frequency and intensity of students' use of artificial intelligence tools in academic tasks. The item pool was generated in line with the theoretical framework of the study and included academic uses of AI such as generating ideas, summarizing content, drafting assignments, editing written work, solving course-related problems, and preparing for exams. The scale consisted of 8 items, and participants responded to each item on a 5-point Likert-type scale ranging from 1 = never to 5 = always. Higher scores on the scale indicated higher levels of AI use in academic contexts. Prior to the main analyses, the wording and content relevance of the items were reviewed, and the psychometric properties of the scale were examined within the present sample.

#### 2.3.6 Academic dishonesty behavior (ADB)

Academic dishonesty was assessed using the Academic Dishonesty Tendency Scale (ADB), the psychometric properties of which were re-examined by Eminoglu Özmercan et al. (2022). The scale was developed to measure tendencies toward academic dishonesty and consists of 22 items and four subdimensions. These subdimensions are defined as Tendency Toward Cheating, Dishonesty Tendency at Studies as Homework, Project, etc.–common, Dishonesty Tendency at Research and Process of Write up, and Dishonesty Tendency Toward Reference. The subdimensions include 5, 7, 4, and 6 items, respectively. The scale is structured in a 5-point Likert-type format, and the response options are completely agree, agree, indecisive, disagree, and completely disagree. Higher scores obtained from the scale indicate higher levels of academic dishonesty tendency. In the relevant study, an internal consistency coefficient of 0.90 was reported for the overall scale, and the four-factor structure of the scale was supported through confirmatory factor analysis. In the present study, the scale was used to assess participants' levels of academic dishonesty.

#### 2.3.7 Social and contextual factors (SCFQ)

Social and contextual factors were assessed using the Social and Contextual Factors Questionnaire (SCFQ) developed by the researchers for this study. The questionnaire was designed to measure the external conditions that may contribute to AI-assisted academic dishonesty. The item pool was constructed on the basis of the conceptual framework of the study and included the dimensions of social norms, peer behaviors, family attitudes, ethical and moral education, insufficient sanctions, teacher attitude, high expectations, and adverse conditions. The instrument consisted of 16 items, and participants responded to the items on a 5-point Likert-type scale ranging from 1 = strongly disagree to 5 = strongly agree. Higher scores indicated stronger contextual conditions associated with academic dishonesty. Prior to the main analyses, the wording and content relevance of the items were reviewed, and the psychometric properties of the questionnaire were examined within the present sample.

To provide additional evidence of construct validity, confirmatory factor analyses were conducted for the measurement instruments used in the study. The standardized factor loadings were examined for each scale, and all retained items demonstrated acceptable loadings. The standardized factor loadings ranged from 0.52 to 0.89 across the instruments, indicating that the observed items adequately represented their intended latent constructs. These findings provided additional support for the construct validity of the measures used in the present sample.

#### 2.3.8 Psychometric validation of researcher-developed instruments

Additional psychometric analyses were conducted for the three researcher-developed instruments used in the present study: the Cheating Tendency Scale, the AI Use Scale, and the Social and Contextual Factors Questionnaire. First, the item pools were reviewed by field experts in educational psychology, measurement and evaluation, and educational technology to assess content relevance, clarity, and conceptual consistency with the theoretical framework of the study. Based on expert feedback, minor wording revisions were made before data collection. Exploratory factor analysis was then conducted to examine the underlying structure of each researcher-developed instrument. The results supported the theoretically expected structures. For the Cheating Tendency Scale, factor loadings ranged from 0.54 to 0.83, and the extracted factors explained 62.47% of the total variance. For the AI Use Scale, factor loadings ranged from 0.61 to 0.88, and the single-factor structure explained 58.36% of the total variance. For the Social and Contextual Factors Questionnaire, factor loadings ranged from 0.57 to 0.86, and the extracted factors explained 65.12% of the total variance. These findings indicated that the retained items loaded sufficiently on their intended dimensions and that the factor structures were consistent with the conceptual framework of the study.

Confirmatory factor analysis was also performed to further evaluate the construct validity of the researcher-developed instruments. The Cheating Tendency Scale demonstrated acceptable model fit, χ^2^/*df* = 2.41, CFI = 0.94, RMSEA = 0.052, and SRMR = 0.046. The AI Use Scale also showed acceptable fit, χ^2^/*df* = 2.18, CFI = 0.96, RMSEA = 0.048, and SRMR = 0.039. Similarly, the Social and Contextual Factors Questionnaire demonstrated adequate fit, χ^2^/*df* = 2.56, CFI = 0.93, RMSEA = 0.055, and SRMR = 0.049. Across the three instruments, standardized factor loadings ranged from 0.52 to 0.89. These results provided evidence that the researcher-developed instruments had acceptable construct validity in the present sample. Internal consistency coefficients were also satisfactory, as reported in Table 1.

**Table 1:** Reliability coefficients, descriptive statistics, and correlations among the study variables.

Variable	α	ω	M	SD	1	2	3	4	5	6	7
1. AP	0.88	0.89	3.12	0.74	—						
2. LH	0.84	0.85	2.96	0.68	0.46^***^	—					
3.ASE	0.91	0.91	4.98	1.01	−0.41^***^	−0.49^***^	—				
4. TC	0.86	0.87	2.71	0.79	0.52^***^	0.55^***^	−0.47^***^	—			
5. AIUS	0.90	0.90	3.64	0.83	0.28^***^	0.24^***^	−0.12^**^	0.39^***^	—		
6. ADB	0.89	0.89	3.08	0.71	0.34^***^	0.31^***^	−0.29^***^	0.49^***^	0.26^***^	—	
7. SCFQ	0.92	0.92	2.38	0.81	0.41^***^	0.44^***^	−0.38^***^	0.63^***^	0.43^***^	0.51^***^	—

AP, academic procrastination; LH, learned helplessness; ASE, academic self-efficacy; TC, cheating tendency; AIUS, AI use scale; SCFQ, social and contextual factors questionnaire; ADB, AI-assisted academic dishonesty; α, Cronbach's alpha; ω, McDonald's omega; M, mean; SD, standard deviation.

^**^p < 0.01.

^***^p < 0.001.

### 2.4 Procedure and data collection

Prior to data collection, the necessary institutional permissions and ethical approval were obtained from the relevant ethics committee. After the approval process was completed, participants were informed about the purpose of the study, the voluntary nature of participation, the confidentiality of their responses, and their right to withdraw from the study at any time without any penalty. Informed consent was obtained from all participants before the administration of the survey. Data were collected during the regular academic semester through an online survey form distributed via institutional communication channels and classroom announcements. The questionnaire package included a personal information form, the Procrastination Assessment Scale–Students (AP), the Learned Helplessness Scale (LH), the Academic Self-Efficacy Scale (ASE), the Cheating Tendency Questionnaire (TC) developed for the present study, and the Academic Dishonesty Tendency Scale (ADB). Participants completed the questionnaires individually, and the average completion time was approximately 25–30 min. To ensure anonymity, no personally identifiable information was collected from the participants. All responses were stored in a secure digital environment and were accessible only to the research team. The data collection process was conducted in accordance with ethical principles related to voluntary participation, confidentiality, and responsible data management.

### 2.5 Preliminary data screening and assumption testing

Before conducting the main analyses, the dataset was screened for missing values, outliers, and statistical assumptions. Since the survey was administered through an online form, incomplete submissions and response patterns with substantial missing data were excluded prior to analysis. Univariate outliers were examined using standardized *z* scores, whereas multivariate outliers were assessed through Mahalanobis distance (Tabachnick and Fidell, 2019). The distributional properties of the variables were evaluated by examining skewness and kurtosis values, together with visual inspection of histograms and Q–Q plots. The findings indicated that the variables met acceptable levels of normality for the planned analyses. Multicollinearity among the predictor variables was assessed using variance inflation factor (VIF) and tolerance values. The VIF values ranged from 1.29 to 2.22, remaining below the commonly recommended threshold of 5, while tolerance values ranged from 0.45 to 0.77, exceeding the minimum acceptable threshold of 0.20. These results indicated that multicollinearity did not pose a serious problem in the dataset. In addition, common method bias was assessed using both Harman's single-factor test and the unmeasured latent method factor approach. Harman's single-factor test was used only as an initial diagnostic procedure. The first unrotated factor explained 31.84% of the total variance, which was below the commonly used 50% threshold. To provide a more rigorous assessment, an unmeasured latent method factor was then added to the measurement model and compared with the baseline model. The inclusion of the common latent method factor produced only a minor improvement in model fit, ΔCFI = 0.006, ΔRMSEA = 0.003, and ΔSRMR = 0.004. In addition, the differences between the standardized factor loadings in the baseline model and the method-factor model were below 0.20. These results indicated that common method variance was unlikely to substantially bias the observed relationships (Podsakoff et al., 2003).

### 2.6 Data analysis strategy

Data analyses were conducted using IBM SPSS Statistics and the PROCESS macro (Version 4.0). Descriptive statistics and Pearson correlation coefficients were calculated for all study variables. Internal consistency reliability was assessed using Cronbach's alpha and McDonald's omega coefficients (Dunn et al., 2014). To test the proposed moderated mediation model, Hayes' PROCESS Model 14 was employed (Hayes, 2018). In these analyses, academic procrastination, learned helplessness, and academic self-efficacy were specified as the independent variables in separate models, cheating tendency was specified as the mediator, AI use was specified as the moderator, and AI-assisted academic dishonesty was specified as the dependent variable. More specifically, the model tested whether cheating tendency mediated the relationship between the psychological antecedents and academic dishonesty, and whether the path from cheating tendency to academic dishonesty was moderated by AI use. Bootstrap resampling with 5,000 samples was applied to estimate indirect effects and generate 95% bias-corrected confidence intervals. Indirect effects were considered statistically significant when the confidence interval did not include zero. In addition, gender, age, and academic grade level were included as control variables in the moderated mediation analyses to account for potential confounding effects. Effect sizes and standardized regression coefficients were reported to facilitate interpretation of the findings.

### 2.7 Ethical considerations

The present study was carried out in accordance with the ethical principles set forth in the Declaration of Helsinki and the regulations of the relevant institutional ethics committee. Prior to the data collection process, ethical approval was obtained from the appropriate university ethics board. All participants were informed about the aim of the study, the voluntary nature of participation, and their right to withdraw from the study at any stage without any negative consequences. Informed consent was obtained from all participants before the administration of the survey. Confidentiality and anonymity were strictly protected throughout the study, and no personally identifiable information was collected. The data obtained were used exclusively for scientific research purposes and were stored securely throughout the research process.

## 3 Results

Results are presented in four stages. First, internal consistency coefficients, descriptive statistics, and bivariate correlations among the study variables are reported. Second, the moderated mediation analyses based on Hayes' PROCESS Model 14 are presented for academic procrastination, learned helplessness, and academic self-efficacy. Third, the conditional indirect effects at different levels of AI use are reported. Finally, the predictive effects of social and contextual factors on AI-assisted academic dishonesty are presented.

### 3.1 Preliminary analyses

Preliminary analyses were conducted before testing the main hypotheses. Internal consistency coefficients were calculated for all measures, followed by descriptive statistics and Pearson correlation analyses to examine the direction and strength of the associations among the study variables. These analyses provided an initial overview of the data structure and the relationships among academic procrastination, learned helplessness, academic self-efficacy, cheating tendency, AI use, social and contextual factors, and AI-assisted academic dishonesty. The results are presented in Table 1.

Table 1 presents the reliability coefficients, descriptive statistics, and bivariate correlations among the study variables. The internal consistency coefficients indicated that all measures demonstrated acceptable to high reliability, with Cronbach's alpha values ranging from 0.84 to 0.92 and McDonald's omega values ranging from 0.85 to 0.92. Among the variables, academic self-efficacy had the highest mean score (*M* = 4.98, *SD* = 1.01), whereas social and contextual factors showed the lowest mean score (*M* = 2.38, *SD* = 0.81). The correlation findings generally supported the proposed hypotheses. Academic procrastination was positively associated with learned helplessness (*r* = 0.46, *p* < 0.001), cheating tendency (*r* = 0.52, *p* < 0.001), AI use (*r* = 0.28, *p* < 0.001), AI-assisted academic dishonesty (*r* = 0.34, *p* < 0.001), and social and contextual factors (*r* = 0.41, *p* < 0.001), while it was negatively associated with academic self-efficacy (*r* = −0.41, *p* < 0.001). Similarly, learned helplessness was positively related to cheating tendency (*r* = 0.55, *p* < 0.001), AI use (*r* = 0.24, *p* < 0.001), AI-assisted academic dishonesty (*r* = 0.31, *p* < 0.001), and social and contextual factors (*r* = 0.44, *p* < 0.001), but negatively related to academic self-efficacy (*r* = −0.49, *p* < 0.001). As expected, academic self-efficacy was negatively correlated with cheating tendency (*r* = −0.47, *p* < 0.001), AI use (*r* = −0.12, *p* < 0.01), AI-assisted academic dishonesty (*r* = −0.29, *p* < 0.001), and social and contextual factors (*r* = −0.38, *p* < 0.001). In addition, cheating tendency showed positive associations with AI use (*r* = 0.39, *p* < 0.001), AI-assisted academic dishonesty (*r* = 0.49, *p* < 0.001), and social and contextual factors (*r* = 0.63, *p* < 0.001).

Finally, AI use was positively related to AI-assisted academic dishonesty (*r* = 0.26, *p* < 0.001) and social and contextual factors (*r* = 0.43, *p* < 0.001), while AI-assisted academic dishonesty was also positively associated with social and contextual factors (*r* = 0.51, *p* < 0.001). Overall, the correlation pattern was consistent with the theoretical model and provided preliminary support for the proposed hypotheses.

### 3.2 Moderated mediation analysis results

To test the proposed moderated mediation model, three separate analyses were conducted using Hayes' PROCESS Model 14. In these analyses, academic procrastination, learned helplessness, and academic self-efficacy were entered as independent variables in separate models, cheating tendency was specified as the mediator, AI use was specified as the moderator, and AI-assisted academic dishonesty was specified as the dependent variable. The analyses examined whether cheating tendency mediated the effects of the psychological antecedents on AI-assisted academic dishonesty and whether the path from cheating tendency to AI-assisted academic dishonesty was contingent upon the level of AI use. The results of these analyses are presented in Table 2.

**Table 2:** Moderated mediation results for the three PROCESS Model 14 analyses.

Path	Model 1: Academic procrastination	Model 2: Learned helplessness	Model 3: Academic self-efficacy
X → M (Cheating tendency)	0.48 (0.04), 12.00^***^	0.51 (0.04), 12.75^***^	−0.43 (0.04), −10.75^***^
M → Y (AI-assisted academic dishonesty)	0.37 (0.05), 7.40^***^	0.35 (0.05), 7.00^***^	0.34 (0.05), 6.80^***^
W → Y (AI use)	0.19 (0.04), 4.75^***^	0.18 (0.04), 4.50^***^	0.20 (0.04), 5.00^***^
M × W → Y	0.14 (0.03), 4.67^***^	0.13 (0.03), 4.33^***^	0.12 (0.03), 4.00^***^
Direct effect of X → Y	0.11 (0.04), 2.75^**^	0.09 (0.04), 2.46^*^	−0.10 (0.04), −2.63^**^
*R^2^* for mediator model	0.27	0.30	0.22
*R^2^* for outcome model	0.46	0.47	0.44
*F* for outcome model	71.28^***^	73.15^***^	66.42^***^

^*^p < 0.05.

^**^p < 0.01.

^***^p < 0.001.

Values are presented as B (SE), t.

X, independent variable; M, cheating tendency; W, AI use; Y, AI-assisted academic dishonesty.

Table 2 presents the moderated mediation analyses conducted using Hayes' PROCESS Model 14. The findings showed that academic procrastination showed a significant positive predictive association with cheating tendency (B = 0.48, SE = 0.04, *t* = 12.00, *p* < 0.001), and a similar positive effect was found for learned helplessness (B = 0.51, SE = 0.04, *t* = 12.75, *p* < 0.001). In contrast, academic self-efficacy significantly and negatively predicted cheating tendency (B = −0.43, SE = 0.04, *t* = −10.75, *p* < 0.001). These findings provided support for H1, H2, and H3. Across all three models, cheating tendency significantly predicted AI-assisted academic dishonesty, indicating that higher levels of cheating tendency were associated with higher levels of dishonest behavior. In addition, AI use significantly predicted AI-assisted academic dishonesty in all models. More importantly, the interaction term between cheating tendency and AI use was significant in each model, showing that AI use strengthened the relationship between cheating tendency and AI-assisted academic dishonesty. These findings supported H4 and H11. The direct effects of academic procrastination, learned helplessness, and academic self-efficacy on AI-assisted academic dishonesty remained significant after the inclusion of the mediator and moderator, suggesting that the observed effects were consistent with a partial moderated mediation pattern. Overall, the results indicated that cheating tendency functioned as an important explanatory mechanism linking the psychological antecedents to AI-assisted academic dishonesty, while AI use intensified the transition from tendency to behavior.

### 3.3 Conditional indirect effects

To further clarify the moderated mediation pattern, the conditional indirect effects of academic procrastination, learned helplessness, and academic self-efficacy on AI-assisted academic dishonesty through cheating tendency were examined at different levels of AI use. Following the conventions of PROCESS Model 14, these effects were estimated at low (−1 SD), mean, and high (+1 SD) levels of the moderator. Bootstrap confidence intervals based on 5,000 resamples were used to determine the statistical significance of the indirect effects. An indirect effect was considered significant when the 95% confidence interval did not include zero. In addition, the index of moderated mediation was examined to determine whether the indirect effect significantly varied across levels of AI use. The results are presented in Table 3.

**Table 3:** Conditional indirect effects and indices of moderated mediation at different levels of AI use.

Model	AI use level	Effect	BootSE	LLCI	ULCI
Model 1: Academic Procrastination → Cheating Tendency → AI-Assisted Academic Dishonesty	Low (−1 SD)	0.09	0.03	0.04	0.16
	Mean	0.15	0.03	0.09	0.22
	High (+1 SD)	0.22	0.04	0.14	0.31
	**Index of moderated mediation**	**0.05**	**0.02**	**0.02**	**0.09**
Model 2: Learned Helplessness → Cheating Tendency → AI-Assisted Academic Dishonesty	Low (−1 SD)	0.10	0.03	0.05	0.17
	Mean	0.16	0.03	0.10	0.23
	High (+1 SD)	0.23	0.04	0.15	0.32
	**Index of moderated mediation**	**0.04**	**0.02**	**0.01**	**0.08**
Model 3: Academic Self-Efficacy → Cheating Tendency → AI-Assisted Academic Dishonesty	Low (−1 SD)	−0.07	0.02	−0.13	−0.03
	Mean	−0.12	0.03	−0.19	−0.07
	High (+1 SD)	−0.18	0.04	−0.27	−0.10
	**Index of moderated mediation**	–**0.04**	**0.01**	–**0.07**	–**0.02**

LLCI, lower limit confidence interval; ULCI, upper limit confidence interval.

Indirect effects were estimated using 5,000 bootstrap samples. An effect was considered statistically significant when the 95% confidence interval did not include zero. Bold values indicate the index of moderated mediation and its corresponding bootstrap standard error and confidence interval.

Table 3 presents the conditional indirect effects of academic procrastination, learned helplessness, and academic self-efficacy on AI-assisted academic dishonesty through cheating tendency at low, mean, and high levels of AI use. For academic procrastination, the indirect effect on AI-assisted academic dishonesty through cheating tendency was significant at all three levels of AI use, and the magnitude of the indirect effect increased from 0.09 at low AI use to 0.22 at high AI use. Similarly, for learned helplessness, the indirect effect was significant across all moderator levels and became stronger as AI use increased, rising from 0.10 at low AI use to 0.23 at high AI use. These findings indicate that the mediating role of cheating tendency became more pronounced when students reported higher levels of AI use.

In contrast, the conditional indirect effect of academic self-efficacy on AI-assisted academic dishonesty through cheating tendency was negative and significant across all levels of AI use. The strength of this negative indirect effect increased from −0.07 at low AI use to −0.18 at high AI use, suggesting that higher academic self-efficacy reduced AI-assisted academic dishonesty through lower cheating tendency, and this protective effect became stronger at higher levels of AI use. Moreover, the indices of moderated mediation were significant in all three models, as their confidence intervals did not include zero. Taken together, these results indicate that AI use significantly conditioned the indirect effects of the psychological antecedents on AI-assisted academic dishonesty through cheating tendency, thereby providing support for the proposed moderated mediation model.

### 3.4 Effects of social and contextual factors on AI-assisted academic dishonesty

To examine the predictive effects of social and contextual factors on AI-assisted academic dishonesty, a multiple regression analysis was conducted. In this analysis, social norms, peer behaviors, family attitudes, ethical and moral education, insufficient sanctions, teacher attitude, high expectations, and adverse conditions were entered as predictors, whereas AI-assisted academic dishonesty was specified as the dependent variable. The results of the regression analysis are presented in Table 4.

**Table 4:** Multiple regression results for the social and contextual predictors of AI-assisted academic dishonesty.

Predictor	B	SE	β	*t*	*p*	VIF
Social norms	0.24	0.05	0.21	4.80	<0.001	1.88
Peer behaviors	0.19	0.05	0.18	4.12	<0.001	1.75
Family attitudes	0.10	0.04	0.09	2.76	0.006	1.42
Ethical and moral education	−0.13	0.04	−0.11	−3.08	0.002	1.37
Insufficient sanctions	0.18	0.05	0.16	3.92	<0.001	1.69
Teacher attitude	0.08	0.03	0.07	2.37	0.018	1.31
High expectations	0.14	0.04	0.12	3.26	0.001	1.54
Adverse conditions	0.21	0.05	0.19	4.35	<0.001	1.73

**Model statistics:** *R*^2^ = 0.39, Adjusted *R*^2^ = 0.38, *F* = 82.14, *p* < 0.001.

B, unstandardized regression coefficient; SE, standard error; β, standardized regression coefficient; VIF, variance inflation factor.

The results presented in Table 4 indicated that the overall regression model was statistically significant, explaining 39% of the variance in AI-assisted academic dishonesty. Among the predictors, social norms (β = 0.21, *p* < 0.001), adverse conditions (β = 0.19, *p* < 0.001), peer behaviors (β = 0.18, *p* < 0.001), and insufficient sanctions (β = 0.16, *p* < 0.001) emerged as the strongest positive predictors of AI-assisted academic dishonesty. In addition, high expectations, family attitudes, and teacher attitude also had significant positive effects on the dependent variable. By contrast, ethical and moral education significantly and negatively predicted AI-assisted academic dishonesty (β = −0.11, *p* = 0.002), indicating that stronger ethical and moral educational experiences were associated with lower levels of dishonest behavior. Overall, these findings suggest that permissive social environments, pressure-related contextual conditions, and weak institutional controls increase the likelihood of AI-assisted academic dishonesty, whereas stronger ethical and moral education functions as a protective factor.

### 3.5 Summary of hypothesis testing

To provide a concise overview of the findings, the results of all hypothesis tests are summarized in Table 5. The table presents each hypothesis together with the corresponding analysis, the key statistical result, and the final decision regarding whether the hypothesis was supported.

**Table 5:** Summary of hypothesis testing.

Hypothesis	Analysis	Key result	Decision
**H1:** Academic procrastination is significantly and positively associated with cheating tendency.	PROCESS Model 14	*B* = 0.48, *p* < 0.001	Supported
**H2:** Learned helplessness is significantly and positively associated with cheating tendency.	PROCESS Model 14	*B* = 0.51, *p* < 0.001	Supported
**H3:** Academic self-efficacy is significantly and negatively associated with cheating tendency.	PROCESS Model 14	*B* = −0.43, *p* < 0.001	Supported
**H4:** Cheating tendency is significantly and positively associated with AI-assisted academic dishonesty behavior.	PROCESS Model 14	Bs = 0.34–0.37, SEs = 0.05, ps <0.001	Supported
**H5:** Academic procrastination is significantly and positively associated with AI-assisted academic dishonesty behavior.	PROCESS Model 14	*B* = 0.11, *p* = 0.006	Supported
**H6:** Learned helplessness is significantly and positively associated with AI-assisted academic dishonesty behavior.	PROCESS Model 14	*B* =0.09, *p* = 0.014	Supported
**H7:** Academic self-efficacy is significantly and negatively associated with AI-assisted academic dishonesty behavior.	PROCESS Model 14	*B* = −0.10, *p* = 0.009	Supported
**H8:** Cheating tendency mediates the association between academic procrastination and AI-assisted academic dishonesty behavior.	Conditional indirect effect	Indirect effect = 0.15, BootSE = 0.03, 95% CI [0.09, 0.22]	Supported
**H9:** Cheating tendency mediates the association between learned helplessness and AI-assisted academic dishonesty behavior.	Conditional indirect effect	Indirect effect = 0.16, BootSE = 0.03, 95% CI [0.10, 0.23]	Supported
**H10:** Cheating tendency mediates the association between academic self-efficacy and AI-assisted academic dishonesty behavior.	Conditional indirect effect	Indirect effect = −0.12, BootSE = 0.03, 95% CI [−0.19, −0.07]	Supported
**H11:** AI use moderates the association between cheating tendency and academic dishonesty behavior; this association is expected to be stronger at higher levels of AI use.	Interaction effect/index of moderated mediation	Bs = 0.12–0.14, SEs = 0.03, ps <0.001; indices of moderated mediation =0.04–0.05, 95% CIs excluded zero	Supported
**H12:** Social norms, peer behaviors, family attitudes, ethical and moral education, insufficient sanctions, teacher attitude, and high expectations are significantly associated with AI-assisted academic dishonesty behavior.	Multiple regression	*R*^2^ = 0.39, *F* = 82.14, *p* < 0.001; strongest predictors were social norms (β = 0.21) and adverse conditions (β = 0.19)	Supported

As shown in Table 5, all proposed hypotheses were supported. Academic procrastination and learned helplessness positively predicted cheating tendency, whereas academic self-efficacy negatively predicted it. Cheating tendency, in turn, significantly predicted AI-assisted academic dishonesty, and the direct effects of the three psychological antecedents on AI-assisted academic dishonesty remained significant. The conditional indirect effects further showed that cheating tendency functioned as a significant mediator in all three models. In addition, AI use significantly moderated the relationship between cheating tendency and AI-assisted academic dishonesty, indicating that the transition from tendency to behavior became stronger at higher levels of AI use. Finally, social and contextual factors significantly predicted AI-assisted academic dishonesty, thereby confirming the explanatory value of the broader socio-contextual framework proposed in the study.

## 4 Discussion and conclusion

The present study offers an integrated interpretation of AI-assisted academic dishonesty as a psychologically grounded, technologically conditioned, and socially embedded behavioral outcome. Rather than indicating that academic dishonesty emerges from a single individual disposition, the findings suggest that dishonest academic behavior in AI-mediated environments is associated with the interplay of psychological vulnerability, cheating-related cognitive readiness, AI use, and contextual conditions. From the perspective of the SOR framework, academic procrastination, learned helplessness, academic self-efficacy, and social-contextual pressures can be interpreted as internal and external stimuli; cheating tendency represents the organismic mechanism through which these conditions are cognitively and motivationally processed; and AI-assisted academic dishonesty represents the behavioral response that may become more likely when AI tools reduce effort costs and blur ethical boundaries. Thus, the main contribution of the study is not merely the confirmation of separate statistical associations, but the demonstration that AI-assisted academic dishonesty should be understood as a socio-technological process in which psychological states, technological affordances, and normative environments operate together. Given the cross-sectional correlational design of the study, these findings should be interpreted as evidence of statistically significant associations rather than causal effects. Therefore, although the tested model is theoretically directional, the results do not establish temporal or causal ordering among the variables.

One of the most consistent findings of the study is that academic procrastination was positively associated with cheating tendency. This result is in line with recent work conceptualizing procrastination as a multidimensional self-regulatory breakdown rather than a simple delay habit. As emphasized by Salguero-Pazos and Reyes-de-Cózar (2023) and Ramadhani et al. (2026), procrastination is closely associated with motivational vulnerability, emotional dysregulation, and deficient self-regulation. The present findings extend this line of research by suggesting that procrastination is not only related to task completion difficulties but may also be associated with cognitive openness to unethical solutions. In digitally mediated environments, where rapid and low-effort alternatives are readily available, procrastination may function as an important risk condition that pushes students toward morally ambiguous shortcuts. This interpretation is also consistent with Rodríguez-Ruiz et al. (2024) and Lei et al. (2025), who suggest that technological affordances can transform procrastination into a more systematic behavioral cycle of avoidance and compensation.

Learned helplessness also showed a significant positive association with cheating tendency, suggesting that a diminished sense of control may be related to students' openness to unethical academic choices. This finding is consistent with the literature showing that learned helplessness weakens persistence, undermines engagement, and reduces effective coping capacity (Azad and Yavuz, 2025; Liu et al., 2024). The present study adds to this literature by suggesting that, in AI-supported learning contexts, helplessness may increase not only passivity but also the tendency to externalize responsibility for academic performance. When students begin to perceive academic success as less dependent on their own effort and more dependent on compensatory tools, they may become more likely to legitimize ethically ambiguous academic conduct. This interpretation resonates with the studies of Zhang et al. (2024) and Acosta-Enriquez et al. (2025), which indicate that low self-regulatory resources and elevated academic stress can direct students toward problematic or substitutive forms of AI use.

By contrast, academic self-efficacy was negatively associated with both cheating tendency and AI-assisted academic dishonesty. This result is theoretically meaningful because self-efficacy reflects not only confidence in performance, but also the capacity to regulate effort, persist under challenge, and maintain adaptive coping strategies. In this respect, the present findings support previous work showing that higher self-efficacy is associated with stronger self-regulation and lower vulnerability to maladaptive academic coping (Acosta-Enriquez et al., 2025; Zhang and Xu, 2025). The findings also reinforce the idea that self-efficacy functions as a psychological filter in technology-rich environments. Students with stronger self-efficacy may be more likely to use AI as a supplementary tool, whereas students with weaker self-efficacy may be more likely to rely on it in a compensatory or dependency-producing way. Thus, the negative association observed in this study suggests that self-efficacy may function as a protective psychological resource associated with lower unethical inclination and lower AI-assisted academic dishonesty in AI-supported academic contexts.

A central contribution of the study lies in demonstrating the mediating role of cheating tendency. The findings indicate that the associations of academic procrastination, learned helplessness, and academic self-efficacy with AI-assisted academic dishonesty were partially mediated by this cognitive-behavioral tendency. This supports the conceptualization of cheating tendency as a proximal cognitive-behavioral mechanism linking internal psychological states with observable academic conduct. Such a pattern is highly consistent with recent behavioral intention approaches in digital learning settings, particularly the work of Huang et al. (2025), who showed that moral evaluations, norm perceptions, and behavioral intentions form the immediate cognitive pathways to dishonest academic action. The present findings therefore strengthen the argument that dishonest behavior is rarely the immediate product of broad psychological characteristics; instead, it is more plausibly explained through narrower cognitive tendencies that bridge predispositions and action.

Another major finding is that AI use significantly moderated the relationship between cheating tendency and AI-assisted academic dishonesty. More specifically, the indirect associations between psychological antecedents and AI-assisted academic dishonesty were stronger at higher levels of AI use. This result is particularly important because it suggests that AI use is not merely parallel to cheating-related tendencies; rather, it may be associated with a stronger link between such tendencies and dishonest academic behavior. This interpretation is closely aligned with the literature portraying AI as a behavioral amplifier. Köbis et al. (2025) argued that delegating tasks to artificial intelligence may reduce the perceived moral cost of unethical action, while Kofinas et al. (2025) and Gonsalves (2025) pointed to the erosion of clear integrity thresholds in AI-enabled academic settings. Similarly, Cotton et al. (2024) emphasized the increasing ambiguity between legitimate assistance and academic misconduct. The present study contributes empirical support to these arguments by showing that the association between cheating tendency and AI-assisted academic dishonesty is stronger among students reporting higher levels of AI use. In other words, the availability, convenience, and functional power of AI tools may create conditions under which pre-existing unethical inclinations are more likely to be associated with dishonest academic behavior.

The findings related to social and contextual factors further reinforce the multilevel nature of academic dishonesty. Social norms, adverse conditions, peer behaviors, and insufficient sanctions emerged as the strongest positive predictors of AI-assisted academic dishonesty, whereas ethical and moral education functioned as a significant negative predictor. These results indicate that academic dishonesty cannot be fully understood in purely individualistic terms. Rather, students appear to interpret and evaluate the ethical acceptability of AI-supported practices within broader normative and institutional environments. This result is strongly consistent with Zanetti and Butera's (2025) collective cheating culture approach, as well as Zhao et al.'s (2025) meta-analytic evidence demonstrating that cultural and normative contexts significantly shape academic cheating behavior. The positive role of peer behavior and social norms is also compatible with the findings of Huang et al. (2025) and Bozkurt et al. (2025), who underline the powerful influence of surrounding practices in normalizing or discouraging dishonest action. At the same time, the negative effect of ethical and moral education suggests that integrity-oriented educational efforts can still serve as an important protective resource, even in technologically complex environments.

From a broader perspective, the study supports the argument that academic dishonesty in the age of artificial intelligence should be conceptualized as a socio-technological phenomenon. The results show that unethical academic behavior emerges not simply from one's personal weakness, but from the intersection of self-regulatory difficulties, vulnerability-related psychological states, cognitive legitimization processes, technological facilitation, and contextual normalization. This integrated pattern is particularly relevant for higher education institutions, where generative AI is becoming increasingly embedded in students' everyday academic routines. The findings imply that institutional responses focused solely on punishment or detection may be insufficient. Reducing AI-assisted academic dishonesty may require a broader strategy that includes strengthening students' self-regulation and self-efficacy, reducing helplessness-producing academic pressures, clarifying institutional guidelines on AI use, and building stronger ethical climates within classrooms and departments.

In conclusion, the present study demonstrates that AI-assisted academic dishonesty is shaped by a dynamic interaction among psychological antecedents, cheating tendency, AI use, and social-contextual conditions. Academic procrastination and learned helplessness were positively associated with cheating tendency, whereas academic self-efficacy was negatively associated with it. Cheating tendency served as a key mediating mechanism, and AI use was associated with a stronger link between this tendency and AI-assisted academic dishonesty. At the same time, the significant effects of social and contextual factors confirm that academic dishonesty is embedded in wider normative and institutional settings. Overall, the findings contribute to the emerging literature on academic integrity in the age of artificial intelligence by offering a comprehensive framework that moves beyond fragmented explanations and highlights the need for psychologically informed, technologically aware, and context-sensitive interventions.

## 5 Limitations and future research

Several limitations of the present study should be acknowledged. First, the study employed a cross-sectional design, which limits the ability to draw causal inferences regarding the relationships among psychological antecedents, cheating tendency, AI use, and AI-assisted academic dishonesty. Although the proposed model was theoretically grounded and statistically supported, the findings should be interpreted in terms of association rather than causation. Future studies could employ longitudinal or experimental designs to examine the temporal and causal dynamics of these relationships more directly. Second, the data were collected exclusively through self-report measures. Although this approach is common in research on academic integrity, self-report data may be affected by social desirability, recall bias, and underreporting, particularly when the topic involves ethically sensitive behaviors. Future research could strengthen the evidence base by incorporating multiple sources of data, such as behavioral indicators, scenario-based measures, instructor evaluations, or digital trace data related to students' actual AI use. Third, although the sample was relatively large and included students from different academic disciplines, the study was conducted within a specific higher education context in Türkiye. Therefore, the generalizability of the findings to other cultural, institutional, and disciplinary settings should be approached with caution. Comparative and cross-cultural studies would be particularly valuable in determining whether the same psychological, technological, and contextual mechanisms operate similarly across different higher education systems. Another limitation concerns the measurement strategy of the study. In addition to established instruments, the study included several researcher-developed measures, particularly for cheating tendency, AI use, and social and contextual factors. While these instruments were developed in line with the theoretical framework of the study and their psychometric properties were examined within the present sample, further validation in independent samples is still needed. Future studies should continue to refine and validate these measures in order to enhance their robustness and broader applicability. Finally, the rapid pace of change in generative artificial intelligence technologies should also be considered a limitation. Students' AI use practices, institutional regulations, and ethical perceptions are evolving quickly, which means that the patterns identified in the present study may shift over time. Future research should therefore examine how changing AI tools, university policies, and instructional practices reshape academic dishonesty in dynamic ways. In particular, intervention-based studies focusing on self-efficacy enhancement, procrastination reduction, ethical education, and AI literacy may provide important practical insights for preventing AI-assisted academic dishonesty in higher education.

## Data Availability

The original contributions presented in the study are included in the article/supplementary material, further inquiries can be directed to the corresponding author.
